# Early clinical outcomes in emergency CABG in the United Kingdom

**DOI:** 10.3389/fcvm.2026.1775479

**Published:** 2026-04-13

**Authors:** Kieran Strong, Kaitlyn Gilbert, Maria Comanici, Denis Ajdarpasic, Jess Donaghue, Jeremy Chan, Gianni Davide Angelini

**Affiliations:** 1 Department of Cardiac Surgery, Bristol Heart Institute, Bristol, United Kingdom; 2 University of Bristol Medical School, Bristol, United Kingdom

**Keywords:** CABG, coronary artery bypass grafting, emergency coronary artery bypass graft (CABG), off-pump coronary aortic bypass grafting, revascualrization

## Abstract

**Introduction:**

There is a paucity of evidence in the literature on trends, revascularisation techniques, and early clinical outcomes for patients undergoing emergency coronary artery bypass grafting (CABG).

**Method:**

All patients who underwent first-time, emergency isolated CABG in the United Kingdom from 1996 to April 2019 were identified from the National Adult Cardiac Surgery Audit database. Trend, early clinical outcomes, and revascularisation technique (on-pump and off-pump) were analysed.

**Results:**

A total of 8,221 patients were included; the median age was 67.9 years and 71% were male. The number of emergency CABG procedures fluctuated over the study period, ranging from approximately 350 cases per year in the early 2000s to around 525 in 2004. Since then, the number has declined, reaching approximately 325 procedures per year. Off-pump coronary bypass (OPCAB) was used in 12% of cases. The overall mortality was 10.9%, and the incidence of return to theatre was 9.4%. Transient ischaemic attack (TIA) occurred in 1.0% of patients, while stroke was observed in 1.7%. The incidence of postoperative dialysis was 8.5%, and deep sternal wound infection occurred in 1.0% of patients. After inverse probability treatment weighting (generating two balanced groups), OPCAB when compared with ONCAB was associated with a lower in-hospital mortality (8.7% vs. 11.2%, *p* = 0.043) and lower incidence of return to theatre (7.3% vs. 9.8%, *p* = 0.035), with no difference in stroke (1.5% vs. 1.7%, *p* = 0.549) and need for post-operative dialysis (8.3% vs. 8.8%, *p* = 0.641).

**Conclusion:**

Emergency CABG remains a high-risk operation with significant in-hospital mortality. The use of OPCAB seems to be associated with lower in-hospital mortality and incidence of return to theatre.

## Introduction and background

Emergency CABG is performed during ongoing myocardial ischaemia when primary percutaneous coronary intervention (PCI) is unsuccessful or not suitable during acute coronary syndrome (ACS) (1). This is reflected in the 2023 European Society of Cardiology (ESC)/European Association of Cardiothoracic Surgery (EACTS) guidelines recommending emergency CABG for patients with a patent infarct-related artery but with unsuitable anatomy for PCI, and either a large myocardial area at jeopardy or with cardiogenic shock. However, in patients presenting with an acute ST- segment myocardial infarction (STEMI) with failed PCI or with an acute coronary occlusion not amenable to PCI, emergency CABG is infrequently performed as the outcomes after surgical revascularisation are less well known (2).

There is limited reporting in the literature on outcomes for patients who underwent emergency CABG, and trials often exclude these patients. Furthermore, controversy persists over the best surgical technique in this situation: off-pump or on-pump coronary artery revascularisation.

This study aimed to explore trends in the use of the surgical technique and early clinical outcomes in emergency CABG over a 23-year period from 1996 to 2019 in the United Kingdom.

## Methodology

All patients undergoing first-time, emergency isolated CABG between 1996 and 2019 were identified from the National Adult Cardiac Surgery Audit (NACSA) database. Individuals who had non-isolated emergency CABG or prior cardiac surgery were excluded. Additionally, cases with missing data regarding cardiopulmonary bypass use were omitted from the analysis. This database prospectively collects data on all major heart operations performed on National Health Service (NHS) in the UK. Details regarding the variable definitions and structure of the NACSA database have been described previously (3). Trends over the 23 years of the study were analysed, including early clinical outcomes and surgical approach.

Emergency surgery was defined as involving unscheduled patients experiencing ongoing, refractory cardiac compromise, for whom surgery or intervention should proceed without delay, regardless of the time of day (4).

Patients were then categorised into two groups: (i) those who underwent on-pump CABG (ONCAB) and (ii) those who underwent off-pump CABG (OPCAB). The two revascularisation techniques were then compared using Inverse probability of treatment weighting (IPTW). The primary endpoint was in-hospital mortality. Secondary end points were in-hospital complications: stroke, return to theatre, requirement for dialysis and deep sternal wound infection (DSWI).

The observed mortality was compared against the expected mortality across all patients. The observed mortality was defined as 30-day or in-hospital mortality after the index operation.

### Ethical statement

This study formed part of an existing research project approved by the Health Research Authority and Health and Care Research Wales. Owing to the retrospective nature of the NICOR database analysis, the need for individual patient consent was waived in line with research governance guidelines (IRAS ID: 278171). The research was carried out in accordance with the ethical principles of the 1964 Declaration of Helsinki and its later revisions.

### Statistical analysis

Normality testing was performed using Shapiro-Wilk test. Continuous variables were expressed as mean (standard deviation, SD) or median (interquartile range, IQR), depending on data distribution, and were compared using Student’s t-test or Wilcoxon rank-sum test, as appropriate. Categorical variables are presented as counts and percentages, with comparisons made using chi-squared exact test.

IPTW was used to adjust for imbalances between patients undergoing OPCAB and ONCAB. Propensity scores were estimated using multivariable logistic regression, which predicts the probability of treatment assignment based on observed covariates (Table 1). These scores were then used to generate weights for each patient; individuals less likely to receive the procedure they underwent were applied a larger weight, and those more likely were assigned a smaller weight. This retains all participants in the analysis and creates a pseudo-population where treatment allocation is independent of confounders, allowing a less biased estimate of the treatment effect.

**Table 1:** Preoperative characteristics of the whole cohort of emergency coronary artery bypass grafting and off-pump and on-pump coronary artery bypass grafting before and after inverse probability treatment weighting (1996–2019).

Preoperative characteristics	Overall	Pre- IPTW			Post- IPTW			
	*n* = 8221	OPCAB (*n* = 997)	ONCAB (*n* = 7224)	*P*-Value	OPCAB (weight=8245)	ONCAB (weight=8221)	SMD	*P*-Value
Age (years), median (IQR)	67.9 (59.3–74.8)	67.5 (59.3–74.6)	68.0 (59.3–74.8)	0.73	67.5 (59.3–74.4)	67.9 (59.2–74.8)	0.0129	0.74
Gender, *n* (%)				0.82			0.0088	0.8
Male	5859 (71.3)	707 (70.9)	5152 (71.3)		5910 (71.7)	8192.3 (71.3)		
Female	2362 (28.7)	290 (29.1)	2072 (28.7)		2335 (28.3)	2361 (28.7)		
BMI, median (IQR)	27.4 (24.5–29.7)	27.4 (24.44–29.91)	27.3 (24.49–29.70)	0.48	27.4 (24.3–29.8)	27.3 (24.5–29.7)	0.0059	0.87
Pulmonary Disease, *n* (%)				0.61			0.0142	0.7
Chronic pulmonary disease requiring use of long-term medication	1018 (12.4)	118 (11.8)	900 (12.5)		982.4 (11.9)	1017.5 (12.4)		
Diabetes, n(%)				0.43			0.0362	0.81
Not diabetic	6335 (77.1)	775 (77.7)	5560 (77.0)		6301 (76.4)	6334 (77.1)		
Diet control	360 (4.4)	35 (3.5)	325 (4.5)		415 (5.0)	360 (4.4)		
Oral therapy	941 (11.5)	110 (11.0)	831 (11.5)		909 (11.0)	941 (11.4)		
Insulin therapy	585 (7.1)	77 (7.7)	508 (7.0)		619 (7.5)	585 (7.1)		
Smoking, n(%)				0.06			0.0115	0.95
Never smoked	2791 (34.0)	317 (31.8)	2474 (34.3)		2832 (34.4)	2791 (34.0)		
Ex-smoker	4043 (49.2)	487 (48.9)	3556 (49.2)		4053 (49.2)	4043 (49.2)		
Current smoker	1387 (16.9)	193 (19.4)	1194 (16.5)		1359 (16.5)	1386 (16.9)		
Peripheral vascular disease, *n* (%)				0.07			0.0033	0.92
Yes	1128 (13.7)	156 (15.7)	972 (13.5)		1140.6 (13.8)	1127.9 (13.7)		
Preop AF, n(%)				0.63			0.005	0.89
Yes	392 (4.8)	44 (4.4)	348 (4.8)		384.4 (4.7)	391.9 (4.8)		
Left main stem (LMS) disease, n(%)				1			0.0037	0.92
LMS > 50% diameter stenosis	12 (0.2)	1 (0.1)	11 (0.2)		10.9 (0.1)	12 (0.2)		
CrCl. Category, n(%)				0.09			0.0303	0.89
Mild	313 (3.8)	30 (3.0)	283 (3.9)		329.0 (4.0)	313 (3.8)		
Moderately Impaired	1053 (12.8)	108 (10.8)	945 (13.1)		1073.4 (13.0)	1053.0 (12.8)		
Normal	6800 (82.7)	851 (85.4)	5949 (82.4)		6766.4 (82.1)	6799.3 (82.7)		
Severely Impaired	55 (0.7)	8 (0.8)	47 (0.7)		75.8 (0.9)	55.5 (0.7)		
CCS class, n(%)				<0.0001			0.0233	0.98
0	922 (11.2)	63 (6.3)	859 (11.9)		949 (11.5)	922 (11.2)		
1	221 (2.70)	25 (2.5)	196 (2.7)		216 (2.6)	221 (2.7)		
2	885 (10.8)	115 (11.5)	770 (10.7)		872 (10.6)	885 (10.8)		
3	1713 (20.8)	250 (25.1)	1463 (20.3)		1651 (20.0)	1712 (20.8)		
4	4480 (54.5)	544 (54.6)	3936 (54.5)		4556 (55.3)	4481 (54.5)		
NYHA class, n(%)				<0.0001			0.022	0.95
1	2443 (29.7)	283 (28.4)	2160 (29.9)		2453 (29.8)	2443 (29.7)		
2	2473 (30.1)	346 (34.7)	2127 (29.4)		2458 (29.8)	2473 (30.1)		
3	1828 (22.2)	245 (24.6)	1583 (21.9)		1900 (23.0)	1829 (22.3)		
4	1477 (18.0)	123 (12.3)	1354 (18.7)		1434 (17.4)	1477 (54.5)		
PCI, n(%)				0.74			0.0124	0.99
No previous PCI	6687 (81.3)	801 (80.3)	5886 (81.5)		6704 (81.3)	6687 (81.3)		
PCI < 24 h before surgery	801 (9.7)	98 (9.8)	703 (9.7)		802 (9.7)	801 (9.7)		
PCI > 24 h before surgery; same admission	215 (2.6)	28 (2.8)	187 (2.6)		231 (2.8)	215 (2.6)		
PCI >24 h before surgery; previous admission	518 (6.3)	70 (7.0)	448 (6.2)		507 (6.1)	518 (6.3)		
Poor mobility, n(%)				0.31			0.0107	0.79
Yes	0.029 (0.17)	0.024 (0.15)	0.030 (0.17)		0.031 (0.17)	0.029 (0.17)		
Ventilated preop, n(%)				0.02			0.0083	0.85
Yes	352 (4.3)	28 (2.8)	324 (4.5)		366.9 (4.5)	351.8 (4.3)		
Cardiogenic shock, n(%)				0.002			0.0186	0.64
Yes	1253 (15.2)	119 (11.9)	1134 (15.7)		1200.8 (14.6)	1251.8 (15.2)		

Prior research has demonstrated the utility of IPTW in achieving covariate balance (5), and Chesnaye et al. (6) highlighted its advantages over propensity score matching, particularly in studies with limited outcome events or many confounders.

**Figure 1:**
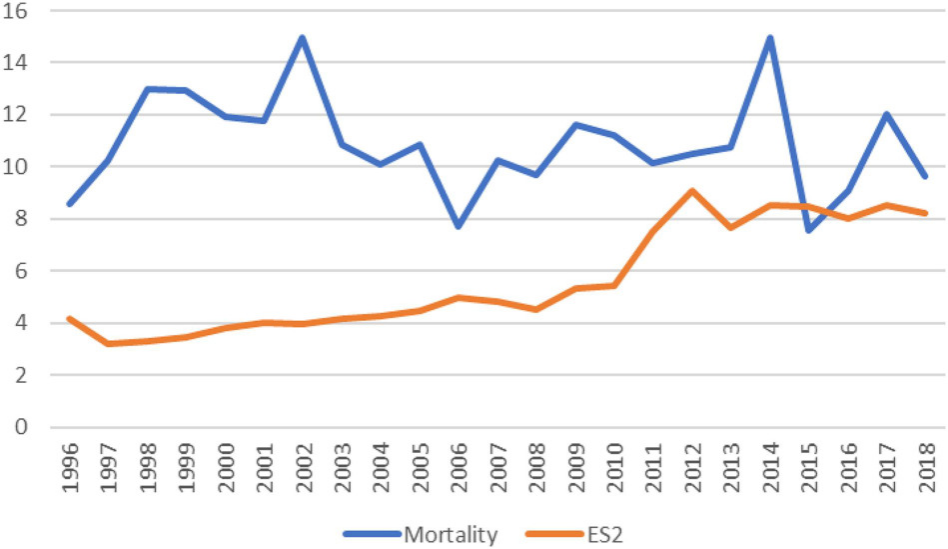
The actual mortality rate (Blue) and the predicted mortality rate (EuroScore II- Orange) for all isolated emergency coronary artery bypass graft from 1996 to 2019.

Covariate balance between the ONCAB and OPCAB groups after IPTW was assessed using standardised mean differences (SMD), with values above 0.10 indicating residual imbalance. Balance before and after weighting is illustrated in Figure 1. Binary logistic regression was performed using the baseline patient demographics and comorbidities to predict factors associated with OPCAB. Results are presented as odds ratio (OR) with 95% confidence interval (95% CI). ONCAB served as the reference category for all comparisons. All statistical analysis were performed in R (Version 4.2.3) and RStudio (Version 1.4.1103, RStudio PBC) using the tidyverse package. Graphs and tables were generated using RStudio and Microsoft Office 365 (Version 16.0.14026, 64-bit). Propensity scores were estimated using multivariable logistic regression including the following preoperative variables: age, sex, body mass index, pulmonary disease, diabetes status, smoking history, peripheral vascular disease, preoperative atrial fibrillation, renal function (creatinine clearance category), left main stem disease, Canadian Cardiovascular Society (CCS) class, New York Heart Association (NYHA) class, previous percutaneous coronary intervention, poor mobility, preoperative ventilation, and cardiogenic shock. Covariate balance before and after weighting was assessed using standardized mean differences (SMD), with values < 0.10 considered to indicate adequate balance between groups.

## Results

### Patients characteristics of the whole cohort

**Figure 2:**
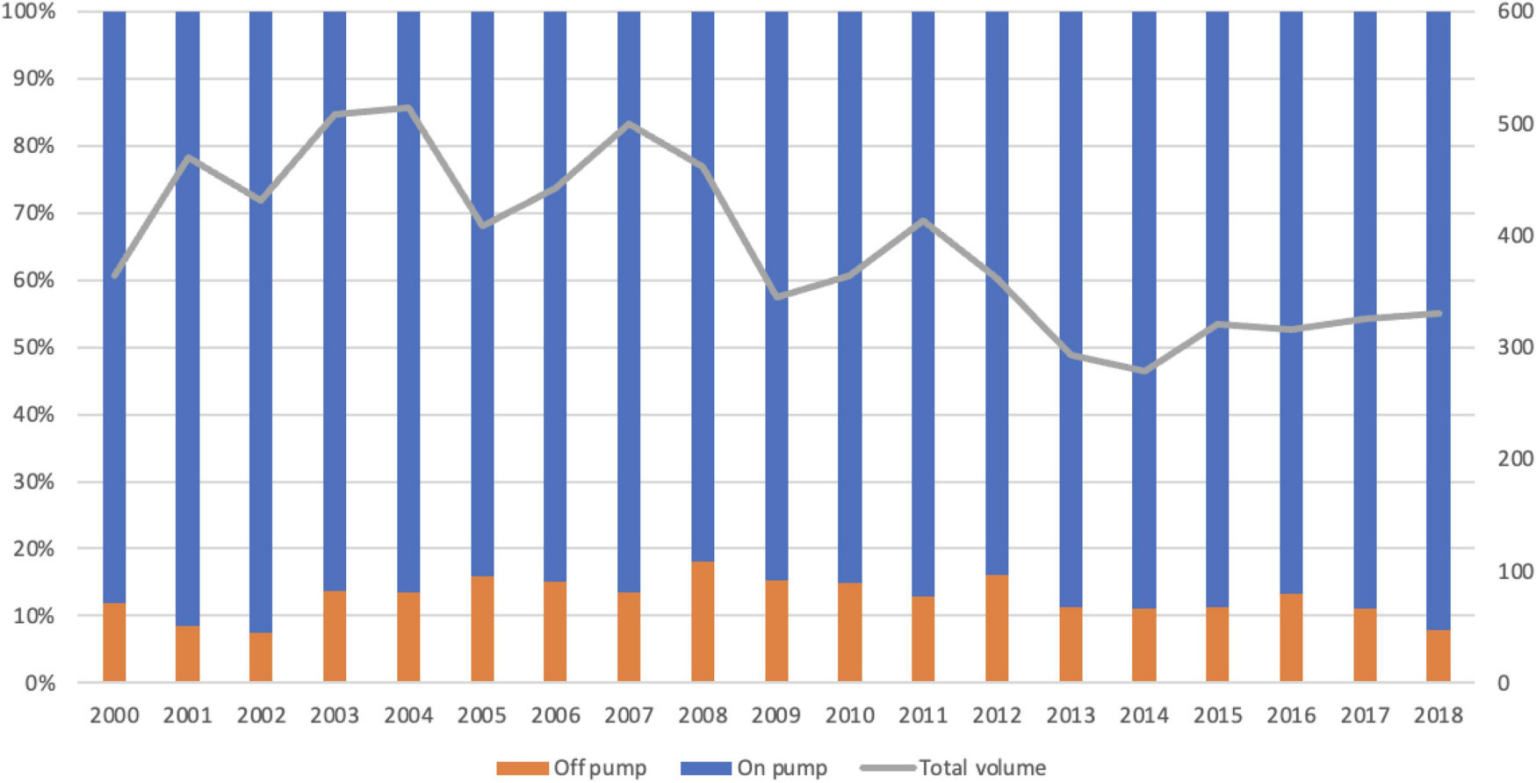
The total number of isolated coronary artery bypass graft (grey) and proportion of emergency on-pump coronary artery bypass graft (blue) and off-pump coronary artery bypass (orange) performed in the UK from 1996 to April 2019.

A total of 8,221 patients were included in the study. The median age was 67.9 years (IQR: 59.3–74.8), 5859 patients (71.3%) were male, and the median body mass index (BMI) was 27.4 (IQR: 24.5–29.7). The number of emergency CABG procedures fluctuated over the study period, ranging from approximately 350 cases per year in the early 2000s to around 525 in 2004. Since then, the number has declined, reaching approximately 325 procedures per year by 2018. A total of 997 (12.1%) underwent emergency OPCAB. The number of OPCAB procedures performed increased in the early 2000s and after peaking in 2008 at 18%, it gradually decreased to 7.88% in 2018 (Figure 2).

### Patients characteristics OPCAB vs. ONCAB after IPTW

**Figure 3:**
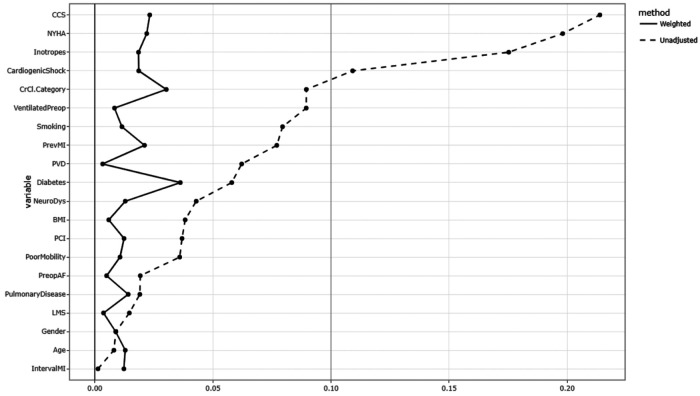
Love Plot depicting the standard mean differences of the baseline covariates before and after inverse probability treatment weighting.

IPTW created two equally weighted groups (weight = 8245, OPCAB and weight=8221, ONCAB) and the preoperative characteristics are shown in Table 1. After IPTW, there was no statistical significance in all variables assessed between OPCAB vs. ONCAB: age (67.5 [IQR: 59.3–74.4 vs.; 67.9 (IQR: 59.2–74.8) years, *P* = 0.74], female gender (28.3% vs. 28.7%, *P* = 0.8) and BMI (27.4 (IQR: 24.3–29.8) vs. 27.3 (IQR: 24.5–29.7),*P* = 0.87 (Table 1). As shown in Table 1 and illustrated in the Love plot (Figure 3), all preoperative covariates achieved satisfactory balance after IPTW, with mean post-weighting SMDs below 0.05 and none exceeding 0.10.

### Intra- and postoperative characteristics of the whole cohort

The mortality rate for all isolated emergency CABG across the study period was 10.9%. The observed mortality rate increased from ∼8% in 1996 to 15% in 2002 and then has been fluctuating back towards 8% in 2018. EuroScore II increased from 4% in 1996 to 8% in 2018. Figure 1 shows the observed and predicted mortality rates for all emergency isolated CABG procedures from 1996 to 2018.The incidence of return to theatre was 9.4% and the incidence of returning to theatre due to bleeding or tamponade was 5.3%. Transient ischaemic attack (TIA) occurred in 1.0% of patients, while stroke was observed in 1.7%. The incidence of postoperative dialysis was 8.5%, and deep sternal wound infection (DSWI) occurred in 1.0% of cases (Table 2).

**Table 2:** The intra- and postoperative outcomes between emergency off-pump and emergency on-pump coronary artery bypass before and after inverse probability treatment weighting (1996–2019).

Intra- and postperative characteristics	Overall	Pre- IPTW			Post- IPTW		
	*n* = 8221	OPCAB (*n* = 997)	ONCAB (*n* = 7224)	*P*-Value	OPCAB (weight=8245)	ONCAB (weight=8221)	*P*-Value
Cardiopulmonary bypass (CPB) time, mean (SD)	N/A	N/A	90.56 (44.70)	N/A	N/A	90.31 (44.49)	N/A
Aortic cross-clamp time, mean (SD)	N/A	N/A	48.31 (30.08)	N/A	N/A	48.18 (29.90)	N/A
Mortality, *n* (%)	890 (10.9)	73 (7.4)	817 (11.4)	<0.001	709.6 (8.7)	913.2 (11.2)	0.043
Return to theatre, n(%)	642 (9.4)	61 (6.8)	581 (9.8)	0.005	545.7 (7.3)	659.1 (9.8)	0.035
Return to theatre with bleeding ortamponade, *n* (%)	438 (5.3)	42 (4.2)	396 (5.5)	0.11	371.2 (4.5)	448.8 (5.5)	0.251
Postop cerebrovascular accident (CVA), n(%)				0.31			0.549
None	6747 (97.3)	865 (98.1)	5882 (97.2)		7125.7 (97.9)	6715.1 (97.2)	
Transient Stroke	72 (1.0)	6 (0.7)	66 (1.1)		47.6 (0.7)	74.7 (1.1)	
Permanent Stroke	115 (1.7)	11 (1.2)	104 (1.7)		108.3 (1.5)	117.8 (1.7)	
Postop dialysis, n(%)	573 (8.7)	63 (7.4)	510 (8.9)	0.155	583.4 (8.3)	574.2 (8.8)	0.641
Postop deep sternal wound infection, n(%)	19 (1.0)	3 (1.4)	16 (1.0)	0.856	19.4 (1.0)	18.5 (1.0)	0.982

### Intra- and postoperative characteristics ONCAB vs. OPCAB after IPTW

The mean CPB and aortic cross-clamp time in the ONCAB group was 90.31 (SD 44.49 min) and 48.18 (SD 29.90), respectively. OPCAB when compared with ONCAB was associated with a lower in-hospital mortality (8.7% vs. 11.2%, *P* < 0.05) and lower incidence of return to theatre (7.3% vs. 9.8%, *P* < 0.05). No differences were seen in the incidence of return to theatre with bleeding and/or tamponade (4.5% vs. 5.5%, *P* = 0.251), postoperative CVA (TIA: 0.7% vs. 1.1%, stroke: 1.5% vs. 1.7%, *P* = 0.55), postoperative dialysis (8.3% vs. 8.8%, *P* = 0.64) and postoperative deep sternal wound infection (1.0% vs. 1.0%, *P* = 0.98) (Table 2).

### Factors predicting the use of OPCAB

The use of OPCAB was more common (but not limited to) in current smokers (OR 0.78, 95% CI 0.64–0.96, *P* < 0.05), patients with peripheral vascular disease PVD (OR 0.81, 95% CI 0.67–0.98, *P* = 0.03), patients with CCS grade 1 (OR 0.55, 95% CI 0.34–0.92), *P* < 0.05), grade 2 (OR 0.49, 95% CI 0.35–0.69), *P* < 0.001), grade 3 [OR 0.43, 95% CI (0.31–0.57), *P* < 0.001], and grade 4 (OR 0.48, 95% CI 0.36–0.64, *P* < 0.001). Patients with NYHA grade 4 (OR 1.50, 95% CI 1.18–1.92, *P* < 0.001) were more unlikely to undergo ONCAB (Table 3).

**Table 3:** The predictors for off-pump coronary artery bypass grafting based on baseline characteristics.

Patient Characteristics	OR (95% CI)	*P*-Value
Age (years)	1.00 (0.99–1.00)	0.18
Gender (female sex)	0.97 (0.84–1.13)	0.72
BMI	0.99 (0.98–1.01)	0.26
Diabetes (diet controlled)	1.32 (0.93–1.93)	0.13
Diabetes (drug)	1.06 (0.86–1.33)	0.59
Diabetes (insulin)	0.91 (0.70–1.19)	0.48
Smoker (ex)	0.98 (0.84–1.15)	0.86
Smoker (current)	0.78 (0.64–0.96)	<0.05
Pulmonary disease	1.09 (0.89–1.36)	0.4
Peripheral vascular disease	0.81 (0.67–0.98)	0.03
Neurological dysfunction	1.80 (0.39–6.11)	0.39
CrCl (mild)	0.96 (0.61–1.46)	0.86
CrCl (moderate)	0.74 (0.49–1.09)	0.14
CrCl (severe)	0.56 (0.25–1.40)	0.18
CCS grade 1	0.55 (0.34–0.92)	<0.05
CCS grade 2	0.49 (0.35–0.69)	<0.001
CCS grade 3	0.43 (0.31–0.57)	<0.001
CCS grade 4	0.48 (0.36–0.64)	<0.001
NYHA grade 2	0.93 (0.77–1.11)	0.42
NYHA grade 3	0.98 (0.80–1.19)	0.84
NYHA grade 4	1.50 (1.18–1.92)	<0.001
PCI <24 h before surgery	0.97 (0.78–1.23)	0.8
PCI >24 h before surgery; same admission	0.87 (0.59–1.33)	0.5
PCI >24 h before surgery; previous admission	0.86 (0.66–1.14)	0.28
Poor mobility	0.73 (0.24–3.19)	0.62
Left main stem disease	1.41 (0.27–26.8)	0.74
Preoperative AF	1.07 (0.78–1.50)	0.7
Ventilated preoperatively	1.07 (0.71–1.65)	0.76
Cardiogenic shock	1.03 (0.82–1.31)	0.77

## Discussion

This large national analysis examined all first-time emergency CABG procedures performed in the UK between 1996 and 2019. It showed that the volume of emergency CABG fluctuated over time but showed an overall decline in recent years. Emergency isolated CABG accounted for 2.3% of all CABG procedures, with annual rates ranging from 1.75% to 3.46%. Off-pump CABG was used in about 12% of emergency cases. The overall mortality rate increased from 1996 to 2002 and subsequently decreased. This is likely to reflect improvements in surgical, anaesthetic techniques and postoperative management. Despite representing a high-risk population with an overall in-hospital mortality of 10.9%, the study found that, after adjusting for baseline differences using inverse probability treatment weighting, OPCAB was associated with significantly lower in-hospital mortality and fewer re-operations compared with on-pump CABG, without differences in stroke, renal failure, or deep sternal wound infection.

The decreasing trend in the volume of emergency and salvage CABGs has been observed worldwide. In a study carried out by Ohri et al. in the United Kingdom between 2002 and 2016, (7) they reported a 33% decrease in emergency CABG over a 15-year period. They also observed a reduction in mortality for emergency CABG (11.7% in 2002–03 to 6.6% in 2015–16), despite higher predicted mortality (18.7% vs. 26%) (7). The STS data illustrated a similar trend with a reduction in emergency and salvage CABG in the United States. In 2005, emergency and salvage CABG constituted nearly 4.9% of all CABGs. This reduced to 4.1% in 2017 (8). Another retrospective Nordic (Sweden, Finland and Iceland) study (between 2006 and 2014) reported a 13% in-hospital mortality for emergency CABG, with the main independent in-hospital mortality risk factors being age, LVEF, pre-operative use of intra-aortic balloon pump, extracardiac arteriopathology, and preoperative use of inotropic drugs (1). Our findings are broadly consistent with this trend, showing a progressive decline in emergency CABG procedures from the early 2000s onward.

The continued decline in emergency CABG volumes in the United Kingdom is likely to be multifactorial, with advances in primary PCI representing the most significant contributor. Current ESC guidelines designate primary PCI as a Class I recommendation for patients presenting with STEMI and cardiogenic shock, effectively reserving emergency surgical intervention for failed or anatomically unsuitable cases (2). PCI has now also been provided with class I recommendation in left main stem stenosis in patients with low syntax scores (9). As reported by Ohri et al. (7), the number of PCI centres in the UK more than doubled from 64 to 119 between 2002 and 2016, coinciding with a parallel rise in PCI rates from 759 to 1,530 per million population. This increasing accessibility and speed of reperfusion have reduced the need for emergency CABG in haemodynamically unstable patients.

Our analysis further examined the comparative outcomes of off-pump vs. on-pump emergency CABG in this high-risk population. In our national analysis, OPCAB was associated with lower in-hospital mortality (8.7% vs. 11.2%, *p* = 0.043) and a reduction in reoperation rates (7.3% vs. 9.8%, *p* = 0.035) compared with ONCAB, findings that align with those of Fattouch et al., who reported reduced reoperation for bleeding and shorter hospital stay in acute STEMI patients treated with OPCAB (10). No significant differences were observed between techniques in postoperative dialysis, cerebrovascular events, or deep sternal wound infection. The 2018 European society of thoracic surgery (ESTS)/EACTS Guidelines on myocardial revascularization recommend that experienced off-pump teams perform OPCAB for high-risk patients (class IIa, level B) (10).

Despite this recommendation the usage of OPCAB in emergency setting continues to be low. Data from the STS database shows that while OPCAB was the strategy for myocardial revascularisation in 17.7% cases between 2005 and 2009, it was only used in 12.8% cases between 2015 and 2017 (11). In our study, the proportion of OPCAB decreased from a peak of 18% in 2008 to a mere 7.8% in 2018. The proportion of OPCAB among non-emergency CABG increased from 1.23% in the early study period to a peak of 19.80%, followed by a gradual decline to 7.63% in the most recent years.

Randomised trials have generally not demonstrated significant differences in short- or long-term mortality between OPCAB and on pump CABG in elective settings (12). However, in emergency settings OPCAB may confer short-term clinical advantages. The safety and favourable long-term outcomes of OPCAB in emergency setting have been confirmed in single centre studies (13). Taken together, these findings suggest that while overall survival equivalence is seen in broader RCT populations, OPCAB may confer short-term clinical advantages in emergency CABG, particularly among high-risk patients who may not tolerate cardiopulmonary bypass or aortic manipulation (12). OPCAB in the UK is mostly confined to specialised units. At present, there are no requirements for training in OPCAB in the national surgical curriculum. Enrolment in a specific fellowship depends on individual trainees; however, there are relatively few such fellowships available in the UK.

## Limitations

This study is subject to several intrinsic limitations. Its retrospective design based on prospectively collected data makes it vulnerable to selection and observational biases. Although inverse probability of treatment weighting (IPTW) was applied to balance covariates, the risk of residual confounding remains. Additional factors such as ejection fraction or regional wall motion abnormalities and the use of preoperative or intraoperative inotropic agents, have not been included in the weighting model due to unavailability in the NACSA database, which could have acted as residual confounders. Furthermore, the OPCAB and ONCAB cohort, changes in perioperative care and surgical techniques over the 23-year study period may have influenced outcomes. Coronary anatomy was not captured in the NACSA database, potentially affecting operative strategy decisions. There is no distinction in the database for on-pump beating CABG, only on pump or off-pump technique. The type of conduit used in emergency CABG procedures has not been reported as part of this study and although this may influence long-term patency, in an emergency capacity, conduit selection is less of a priority due to the high mortality rate in these patients. The number of grafts per patient has not been included but is unlikely to have influenced the surgical technique used. Mortality data in our study has been reported as the ‘observed mortality' for the cohort and no comparisons have been made comparing ‘observed to expected' mortality.

There is some missing data as the NACSA database relies on human factors, it is the clinician's role to input data, but it is not mandatory to record all observations and post operative outcomes. Although OPCAB conversion is known to increase perioperative risk, the conversion rate in our cohort was not detailed due to the data being omitted although we believe this to have a minimal impact on the overall results. There is also a lack of long term follow up data to further analyse longer-term outcomes i.e., patency, revascularisation, and 5-year survival rate. Despite these limitations, we believe this study contributes meaningful insights to the ongoing discussion about procedural selection and outcomes in emergency coronary surgery.

## Conclusion

Our data demonstrate a declining trend in the total number of emergency CABG procedures performed each year. This reduction is likely multifactorial, primarily reflecting advances in PCI and increasing patient comorbidity. Mortality remains high at 10.9%. Notably, there has been a marked decline in the use of emergency OPCAB since 2008. The use of OPCAB, appears to be associated with lower in-hospital mortality and incidence of return to theatre.

OPCAB may offer an effective alternative method of revascularisation in emergency CABG, particularly for high-risk patients who may not tolerate the physiological burden associated with a conventional on-pump procedure.

## Data Availability

The original contributions presented in the study are included in the article/Supplementary Material, further inquiries can be directed to the corresponding author.
